# Spillover Effects When Taking Turns in Dyadic Coping: How Lingering Negative Affect and Perceived Partner Responsiveness Shape Subsequent Support Provision

**DOI:** 10.3389/fpsyg.2021.637534

**Published:** 2021-03-04

**Authors:** Lisanne S. Pauw, Suzanne Hoogeveen, Christina J. Breitenstein, Fabienne Meier, Valentina Rauch-Anderegg, Mona Neysari, Mike Martin, Guy Bodenmann, Anne Milek

**Affiliations:** ^1^Couple and Family Psychology Lab, Department of Psychology, Münster University, Münster, Germany; ^2^Department of Social Psychology, Faculty of Social and Behavioral Sciences, University of Amsterdam, Amsterdam, Netherlands; ^3^Clinical Psychology for Children/Adolescents and Couples/Families, Department of Psychology, University of Zürich, Zurich, Switzerland; ^4^University Research Priority Program Dynamics of Healthy Aging, Department of Psychology, University of Zürich, Zurich, Switzerland

**Keywords:** dyadic coping, support provision, perceived responsiveness, negative affect (NA), interpersonal emotion regulation, behavioral observation

## Abstract

When experiencing personal distress, people usually expect their romantic partner to be supportive. However, when put in a situation to provide support, people may at times (still) be struggling with issues of their own. This interdependent nature of dyadic coping interactions as well as potential spillover effects is mirrored in the state-of-the-art research method to behaviorally assess couple’s dyadic coping processes. This paradigm typically includes two videotaped 8-min dyadic coping conversations in which partners swap roles as sharer and support provider. Little is known about how such dyadic coping interactions may feed back into one another, impacting the motivation and ability to be a responsive support provider. In three behavioral studies, we examined how sharers’ experiences may spill over to affect their own support provision in a subsequent dyadic coping interaction. We hypothesized that the extent to which sharers perceive their partner as responsive to their self-disclosure increases the quality of their own subsequent support provision (Hypothesis 1), whereas sharers’ lingering negative affect reduces the quality of their own subsequent support provision (Hypothesis 2). In line with our first hypothesis, perceived partner responsiveness predicted the provision of higher-quality support, though primarily as perceived by the partner. Sharers who perceived their partner to have been more responsive were somewhat more likely to subsequently engage in positive dyadic coping and were rated as more responsive by their partners. Negative dyadic coping behavior was unaffected. Evidence for our second hypothesis was mixed. While lingering negative affect did not affect positive dyadic coping behavior or perceived support, it did increase the chances of negative dyadic coping behavior. However, given the very low occurrences of negative affect and negative dyadic coping, these findings should be interpreted with caution. Taken together, these findings suggest that support interactions may feed back into one another, highlighting the complex and interdependent nature of dyadic coping. The strongest and most consistent findings concerned the spillover effect of perceived partner responsiveness on subsequent perceived support quality, speaking to the key role of *believing* that one’s partner is responsive to one’s needs in promoting healthy relationship functioning.

## Introduction

Imagine coming home after a rough day at work, where your boss unexpectedly just fired one of your favorite colleagues. Your partner gives you a hasty kiss, after which they fires away with a long story on their concerns about his or her mother’s deteriorating health. How do you respond? While you might normally be a very attentive listener, trying to gauge his or her needs to best support him or her, you may find yourself preoccupied with lingering anger, sadness, and confusion about your colleague’s dismissal. Also, you might perceive your partner to be unresponsive, not sensing your current mood, or asking about your day, but simply showering you with his or her own concerns. As a result, you may find yourself unable or unwilling to be a responsive partner in this situation.

One of the core features of close relationships concerns sharing one’s intimate emotional experiences with one’s partner (e.g., Laurenceau et al., 2004). This type of self-disclosure is crucial for fostering intimacy and hinges on the partner’s responsiveness to sharer’s needs (for overviews, see Laurenceau et al., 2004; Reis et al., 2004, 2017; Reis and Gable, 2015). The process of dyadic coping describes how one partner’s emotional expression allows the couple to evaluate the nature and implications of the distressing situation together, paving the way for support provision (i.e., dyadic coping, see systemic transactional model; Bodenmann, 1995; Bodenmann et al., 2016). Positive dyadic coping, which includes attentive listening and various forms of emotional (e.g., empathy), cognitive (e.g., reappraisal), and instrumental support (e.g., practical assistance), has been shown to be crucial for relationship satisfaction (see a meta-analysis by Falconier et al., 2015). However, as illustrated in the scene above, when one’s own support provision is requested, people may at times (still) be struggling with issues of their own. An overlooked issue is how these support interactions may feed back into one another, impacting the quality of support provision. This interdependent nature of dyadic coping interactions as well as potential spillover effects is mirrored in the state-of-the-art research method to behaviorally assess couple’s dyadic coping processes. This paradigm typically includes two videotaped 8-min dyadic coping conversations in which partners swap roles as sharer and support provider. These interactions are typically studied as independent while they likely are not (see Laurenceau et al., 2004; Joseph and Afifi, 2010; Joseph et al., 2016; Leuchtmann et al., 2018).

In three behavioral studies, we examine whether and how the nature of a preceding dyadic coping interaction shapes support provision in a subsequent interaction. Several cognitive and motivational factors have been theorized to shape how romantic partners provide support, some of which may be more global (e.g., problem-solving skills or relationship satisfaction) and others more situational (e.g., current available resources or evaluations regarding the need for support; Bodenmann, 1995; Falconier et al., 2015; Bodenmann et al., 2016). The extent to which sharers still experience lingering negative affect and the perceived responsiveness of their partner might constitute two such situational factors that may impact their (cognitive) ability and motivation to provide support when acting as a listener in the next conversation. More specifically, we hypothesize that the extent to which sharers perceive their partner to have been responsive to their self-disclosure of a personal stressor increases the quality of their own subsequent support provision, whereas the sharers’ lingering negative affect reduces the quality of own their subsequent support provision. Reflecting potential interdependence between two subsequent dyadic coping interactions, these hypothesized dynamics have methodological implications for the conclusions that can be drawn from data relying on this paradigm, as well as broader theoretical implications for support interactions in daily life.

### Spillover Effects of Perceived Partner Responsiveness

When experiencing personal distress, people usually expect their romantic partner to be supportive (Clark et al., 2001; Feeney and Collins, 2001; Reis et al., 2004; Hampel and Vangelisti, 2008)). When partners respond to this distress in a way that makes sharers feel validated, understood, and cared for, sharing interactions may foster perceived partner responsiveness, which has been defined as “the process by which individuals come to believe that relationship partners both attend to and react supportively to central, core defining features of the self” (Reis et al., 2004, p. 203). A wealth of literature has shown that when people experience their partners as being responsive to their emotional disclosures, they feel better, more secure in the relationship, and closer to their partner (Laurenceau et al., 1998; Feeney and Collins, 2001, 2003; Manne et al., 2004; Reis et al., 2004; Lemay et al., 2007; Maisel and Gable, 2009; Kuhn et al., 2018; Pagani et al., 2019). Conversely, when people perceive their partner to be less responsive than desired, they experience greater negative affect, reduced positive affect, and reduced relationship satisfaction (Siewert et al., 2011; Afifi et al., 2013; Priem and Solomon, 2015; Joseph et al., 2016).

Crucially, these emotional and relational outcomes of support interactions likely set a cyclical dynamic in play. When people perceive their partner to be responsive, they may be motivated to reciprocate this benevolence, to be compassionate and responsive to their partner when the tables turn and they themselves are put in a situation to provide support (see Reis, 2014). Most pieces of evidence for such upward spirals of perceived partner responsiveness shaping enhanced pro-relational behavior come from studies examining these dynamics on a trait level over longer periods of time (e.g., Wieselquist et al., 1999; Feeney and Collins, 2003; Lemay and Clark, 2008). However, one set of studies supports the cyclical nature of responsiveness and compassionate motivation, showing that when people perceived their roommate to be more responsive, they experienced greater compassionate goals, which in turn predicted greater reciprocal responsiveness toward their roommate (Canevello and Crocker, 2010). Together, these studies point to the dynamic nature of support provision and suggest that one partner’s experiences as a sharer may shape his or her own support provision when the roles are reversed.

### Spillover Effects of Negative Affect

Another consequence of this dynamic interplay of switching between seeking support and providing support may be that personal stressors cause lingering negative affect to spill over into the next support interaction. Such sequences of stress expression are likely to occur in couples’ lives on a regular basis (e.g., when both partners come home from work) and are also reflected in the frequently adopted methodological paradigm in which couples engage in two subsequent dyadic coping interactions. Spillover effects of negative affect are particularly likely to occur when people have recently shared their emotional experience, as discussing one’s own emotional experience reactivates and prolongs the emotional experience (Rimé, 2009; Verduyn et al., 2009, 2011). Furthermore, negative affect might even be increased when people perceive their partner as unresponsive to their sharing (e.g., Joseph et al., 2016). Consequently, the (lingering) experience of negative emotions—whether due to the personal stressor or the sharing experience with one’s partner—may impede people’s ability and motivation to provide responsive support to one’s partner in several ways.

First, emotional arousal may reduce cognitive abilities that are necessary for being there for one’s partner, for example, by attentive listening, perspective taking, or accurately perceiving his or her emotions (see Epley et al., 2004; Israelashvili et al., 2020a). Negative emotional experiences might trigger a ruminative process in which people keep thinking about the negative emotional experience (Curci et al., 2013). This ruminative process impairs working memory capacity that would otherwise be available for attending responsively to one’s partner (Curci et al., 2013; English and John, 2013) or for downregulating one’s own emotions (Schmeichel et al., 2008; Raio et al., 2013; Schmeichel and Tang, 2015). Particularly expressive suppression, that is, trying not to show one’s feelings (in this case, to one’s partner), has been shown to be cognitively demanding (e.g., Webb et al., 2012; Franchow and Suchy, 2015). Consequently, trying to suppress one’s emotions may distract one from attending to one’s interaction partner, which may result in behavior that seems distracted or uninterested (i.e., superficial dyadic coping; Bodenmann, 2005). In line with this idea, Butler et al. (2003) showed that those who suppressed their negative emotions (compared to those who did not) engaged in less responsive behavior, which led their partners to feel less close to them (see English et al., 2013).

Furthermore, preoccupation with one’s own negative emotions may elicit overarousal in response to one’s partner’s negative emotions, causing personal distress (Eisenberg and Eggum, 2011). Personal distress impairs the ability to accurately gauge one’s partner’s emotions (Israelashvili et al., 2020b) and may induce a primary motivation to reduce one’s own distress rather than one’s partner’s distress (see Eisenberg and Eggum, 2011). Such self-focused caregiving motivations have been found to be associated with ineffective forms of support, in contrast to more altruistic motivations, which are associated with more responsive caring (Feeney and Collins, 2001, 2003). Further supporting this notion, prior work shows that the experience of greater personal distress is associated with a reduced motivation to be compassionate toward others (Crocker et al., 2010), decreased emotional and instrumental support, and greater negative dyadic coping behavior (e.g., criticizing, inattention, disengagement, unhelpful advice; Devoldre et al., 2010; Iida et al., 2010). Taken together, the experience of lingering negative affect may thus temporarily impair both the ability and motivation to be a responsive support provider.

### Overview of the Present Research

Romantic relationships are characterized by the dynamic, dyadic nature of their efforts to cope with emotional distress, with partners continuously switching between the roles of sharer and support provider. While a wealth of research demonstrates the benefits of obtaining responsive support, it remains relatively elusive what predicts whether partners will *provide* responsive support (see Canevello and Crocker, 2010; Crocker et al., 2010; Collins et al., 2014). Considering exactly this dynamic interplay of dyadic coping, the present set of studies aimed to examine how the nature of a (prior) support-seeking experience shapes the motivation and ability to provide responsive support when roles are reversed. Hereby, we focused on potential spillover effects of two key factors: perceived partner responsiveness and lingering negative affect. More specifically, we hypothesized that the extent to which sharers perceived their partner to have been responsive in a first dyadic coping interaction increases the quality of their own subsequent support provision (Hypothesis 1), whereas lingering negative affect of the sharer after the first dyadic coping interaction reduces the quality of his or her own subsequent support provision (Hypothesis 2). It should be noted that the present research focused solely on sharers disclosing a personal stressor that is unrelated to the partner.

To test these hypotheses, we present three behavioral studies (total *N* = 728 male–female couples) in which romantic partners engaged in two subsequent videotaped 8-min dyadic coping interactions. In the first interaction, one partner started as a sharer, telling his or her partner about a stressful experience external to the relationship. Roles were swapped in the second conversation. Quality of support was assessed in three different ways. First, we examined the quality of support as perceived by the partner (i.e., perceived responsiveness; Reis and Gable, 2015). Second, we included two behavioral measures of support quality: the frequency of positive and negative dyadic coping behavior, reflecting high and low quality of support, respectively. Observed behavior was coded by trained coders with a well-established coding system (i.e., Coding System for Dyadic Coping; Bodenmann, 2000). Studies 2 and 3 served as replications of Study 1. All hypotheses and analyses were preregistered on OSF (see here for Study 1 and here for Studies 2 and 3).

## Study 1

### Methods

#### Participants

The data of Study 1 were part of a longitudinal study including 11 waves (Bodenmann et al., 2019). This research project examines couples’ transition to parenthood and included a randomized controlled trial for two couple-focused interventions. The current dataset constituted the first wave of this project, in which participants had not received any intervention yet and were in the third trimester of pregnancy with their first child. Recruitment took place by distributing leaflets or approaching expecting couples directly in different hospitals, gynecological practices, and pregnancy yoga courses, as well as through different social media platforms, newspaper ads, and newsletters. Eligibility criteria included (1) being in a committed romantic relationship of at least 1 year, (2) the female being up to 27 weeks pregnant of their first child, (3) both partners agreeing to participate in the study, (4) understanding and speaking German, and (5) not currently being in treatment for physical or psychological illness. A total of 284 mixed-gender couples took part in Study 1.

As described in our preregistration (see here), we excluded participants from the analyses when they had a predefined number of missing values on the variables that were relevant for that particular analysis. Since most of our measures were averaged composite scores, as a standard, predefined rule across all our three studies, we included participants who had valid data for at least two thirds of the items or video segments per construct. For 34 couples, we did not have behavioral (video) data due to technical reasons and some couples not giving permission to use their video data. This resulted in a total sample of 236 couples for the analyses predicting positive and negative dyadic coping and 262 couples for the analyses predicting perceived responsiveness. On average, women were 31.9 years old (*SD* = 3.6, range = 21–42 years) and men were 34.0 years old (*SD* = 5.1, range = 23–63 years). Most participants reported a relationship duration of 1–5 years (∼45%) or 5–15 years (∼52%). Most of the couples were married (55.4%), and almost all couples (98.2%) were cohabiting.

#### Procedure

**FIGURE 1:**
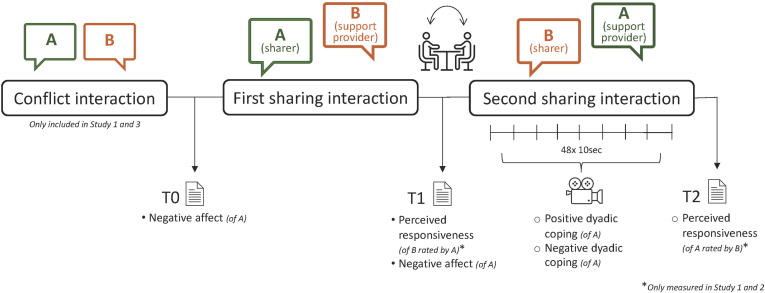
Graphical depiction of the experimental procedure across the three studies.

After providing informed consent, participants first filled out an online questionnaire about their relationship (including other constructs that are beyond the scope of the current study but can be found here in the study protocol). Next, couples were visited at home, where they took part in three videotaped interactions. First, they had a conflict interaction (irrelevant to the present study) after which they engaged in two dyadic coping interactions. In the first dyadic coping interaction, one partner was randomly assigned the role of the sharer. Before the conversation, sharers rated the extent of burden they experienced in response to a list of topics external to the relationship. Sharers were then asked to talk about the most burdensome topic that still affected them (e.g., that they were still thinking or feeling bad about) that was not directly associated with the partner or the relationship (i.e., an external stressor) and they felt comfortable discussing in front of the camera. The support provider was not instructed to respond in a certain way. Both were instructed to behave in a way they usually do (apart from being asked not to leave the room). In the second dyadic coping interaction, the roles were reversed. After each interaction, sharers rated their negative affect and the extent to which they perceived their partner to have been responsive throughout the conversation. The procedure is visually displayed in Figure 1. The study was approved by the ethics committee of the Department of Psychology of the University of Zurich.

#### Materials

##### Negative affect

Before (T0) and after (T1) the first dyadic coping interaction, sharers rated the extent to which they experienced nine negative emotions (i.e., unwell, distressed, bad, annoyed, angry, agitated, anxious, restless, stressed, sad) on a 5-point Likert scale ranging from 1 (not at all) to 5 (very much). These items were averaged to reflect a pre- and post-sharing negative affect composite score.

##### Perceived responsiveness

After each dyadic coping interaction, sharers rated the extent to which they perceived their partner to have been responsive. More specifically, sharers rated the following two items on a 5-point Likert scale ranging from 1 (not at all) to 5 (very much): “In the conversation with my partner, I felt supported” and “In the conversation with my partner, I felt understood.” An average was used as an indicator of perceived responsiveness, with the measure after the first dyadic coping interaction (T1) serving as a key predictor and the measure after the second dyadic coping interaction (T2) serving as a dependent variable.

##### Positive and negative dyadic coping

As behavioral measure of support quality, we examined the frequency of positive and negative dyadic coping behaviors using a well-established coding system (Coding System for Dyadic Coping; SEDC; Bodenmann, 2000). Coders were trained to a criterion of 0.90 on interrater agreement, assessed by Cohen’s kappa, requiring a minimum of 60 h of coding. Each video was coded by two coders who focused on either partner. Each 8-min interaction was divided into 48 sequences of 10 s each, which were coded for the presence (1) or absence (0) of various positive and negative dyadic coping behaviors. Proportion scores for positive and negative dyadic coping were calculated over the total number of validly coded 10-s sequences. This thus resulted in two final individual scores ranging from 0 to 1, with 0 reflecting no positive (negative) dyadic coping at all and 1 reflecting positive (negative) dyadic coping throughout the entire conversation.

Positive dyadic coping was composed of three subcategories: attentive listening, problem-focused dyadic coping, and verbal emotion-focused dyadic coping. Attentive listening required the support provider to be oriented toward the sharer and make eye contact and also included nodding, backchanneling (e.g., “mmm,” “yeah”), and reinforcing questions about the sharer’s emotional experience (e.g., “How did that make you feel?”). Problem-focused dyadic coping included any attempt to help solve the problem, such as giving advice or providing assistance in dealing with the problem (e.g., “Maybe you could try to work a bit more slowly next time”). Emotion-focused dyadic coping included any attempt to help the partner cope with the emotions elicited by his or her problem, such as by conveying understanding and validation (e.g., “I understand this must be difficult for you”), expressing faith in his or her partner (e.g., “I know you can do it”), and helping to reappraise the situation (e.g., “I understand that this is bad for you, but if you see the whole thing in a bigger context, it is not as important as it seems at first glance”).

Negative dyadic coping consisted of any support behavior that was hostile, ambivalent, dismissive, or superficial. These negative forms of dyadic coping could be manifested verbally, such as by sarcastic or critical responses to the partner’s stress expression, or nonverbally or para-verbally, such as when a verbally supportive response was accompanied by a disinterested face or tone of voice, averted gaze, or posture.

#### Data Analytic Approach

##### Statistical models

In all our models, we included sharers’ negative affect before the first dyadic coping interaction (T0) as a control variable and negative affect after the first dyadic coping interaction (T1) and perceived partner responsiveness rated after the first dyadic coping interaction (T1) as key predictors of their subsequent support quality when acting as a support provider in the second dyadic coping interaction (T2). It should also be noted that we ran several supplemental and exploratory analyses as specified in our preregistrations. These are reported in the Supplementary Material and included controlling for relationship satisfaction and stress expression of the partner in the second dyadic coping interaction and the examination of any potential moderation effects by gender, potential interaction effects between our key predictors, and any potential effects of the experimental order of the two dyadic coping interactions. All predictors were centered, and missing values were removed (separately for each dependent variable) before entering them in the analyses.

To account for the fact that positive and negative dyadic coping behavior were quantified as proportions scores, including zeros (behavior is never displayed) and ones (behavior is always displayed), we used zero-one-inflated beta regression models for the analyses predicting dyadic coping. Specifically, we used the R package brms (Bürkner, 2017) to fit the Bayesian zero-one-inflated regression models and the R package BayesFactor (Morey et al., 2018) to fit standard Bayesian linear regression models predicting perceived support. In the Bayesian framework, evidence is quantified by means of a Bayes factor that reflects the extent to which the data support the presence vs. absence of the effect of interest. We expected a directed effect for all specified hypotheses (i.e., a one-sided test) and thus adjusted the Bayes factors accordingly. For each hypothesis, a Bayes factor BF_10_ was calculated. The subscripts on the Bayes factor refer to the hypotheses being compared, with the first subscript referring to the one-sided hypothesis of interest (i.e., a positive or negative effect) and the second subscript referring to the null hypothesis. For instance, BF_10_ = 2 indicates that the data are two times more likely under the alternative hypothesis that there is a (positive or negative) effect than under the null hypothesis that there is no effect. Notably, the Bayesian paradigm allows one to distinguish between “absence of evidence” (i.e., the data are uninformative regarding the absence or presence of an effect; BF_10_ = 1) and “evidence of absence” (i.e., evidence in favor of the null hypothesis that there is no effect, or put differently, evidence *against* an effect; BF_10_ < 1). As the evidence is quantified on a continuous scale, we also present the results as such. Nevertheless, we included a verbal summary of the results by means of the interpretation categories for Bayes factors proposed by Lee and Wagenmakers (2013) based on the original labels specified by Jeffreys (1939). As a rough guideline, we consider Bayes factors larger than 10 as compelling evidence for the effect of interest, Bayes factors between 3 and 10 as weak to moderate evidence for the effect, Bayes factors between 1/3 and 3 as no to weak evidence, and Bayes factors smaller than 1/3 as weak to moderate evidence *against* the effect of interest.

##### Prior specification

As we expected modestly sized effects, we used weakly informative prior distributions for the key predictors in the zero-one-inflated beta models (i.e., a normal distribution with a mean of 0 and a standard deviation of 0.5). For the zero-one-inflated beta regression models, default priors in brms were used for the intercepts. These include a Student-*t* prior with 3 degrees of freedom, a mean of 0 and a scale of 2.5 on the overall intercept, and a logistic(0,1) prior on the intercepts for the zero-one inflation and conditional-one inflation. For the normal linear regression models, the default settings in the BayesFactor package were used (Rouder and Morey, 2012; Rouder et al., 2012), that is, a Cauchy prior with a scale of $\frac{\sqrt{2}}{4}\approx$ 0.35 on the key predictors.

### Results

#### Spillover Effects of Perceived Partner Responsiveness

**FIGURE 2:**
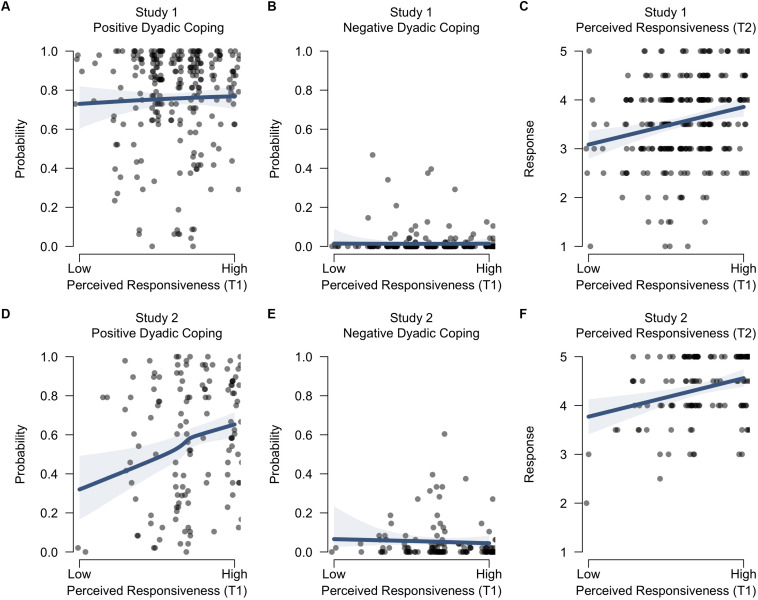
Predicted support quality during the second interaction as a function of perceived responsiveness during the first dyadic coping interaction. The left panels **(A,D)** display the effects on positive dyadic coping behavior (as observed), the middle panels **(B,E)** on negative dyadic coping behavior (as observed), and the right panels **(C,F)** on perceived responsiveness (as rated by the partner at T2). The top row shows the effects for Study 1 and the bottom row for Study 2. The shaded bands reflect the 95% credible interval, and the circles are observed data points. The circles are slightly horizontally jittered to enhance visibility.

**FIGURE 3:**
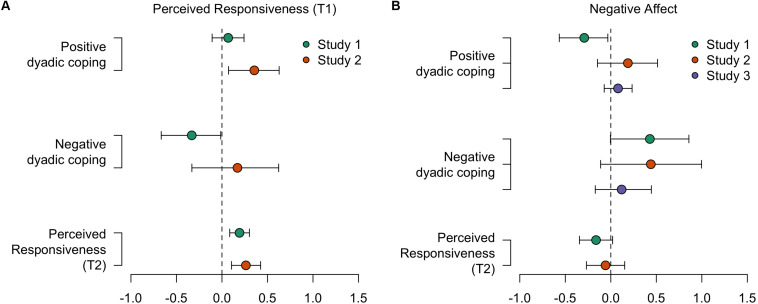
Estimated effects of lingering negative affect after the first dyadic coping interaction **(A)** and of perceived responsiveness during the first interaction **(B)**. For positive and negative dyadic coping, effects are displayed on a logit scale. For perceived responsiveness (T2), effects are displayed on the response scale (1–5).

**FIGURE 4:**
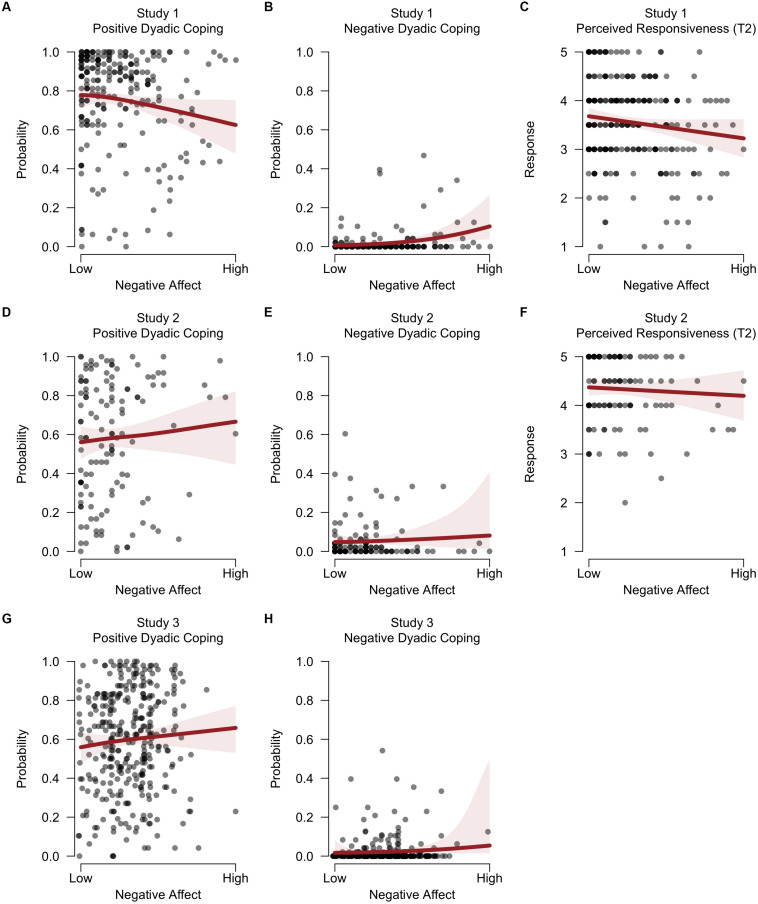
Predicted support quality during the second dyadic coping interaction as a function of lingering negative affect after the first dyadic coping interaction. The left panels **(A,D,G)** display the effects on positive dyadic coping behavior (as observed), the middle panels **(B,E,H)** on negative dyadic coping behavior (as observed), and the right panels **(C,F)** on perceived responsiveness (as rated by the partner at T2). The top row shows the effects for Study 1, the middle row for Study 2, and the bottom row for Study 3. The shaded bands reflect the 95% credible interval, and the circles are observed data points. The circles are slightly horizontally jittered to enhance visibility.

With regard to the first hypothesis, we assessed the evidence for an effect of perceived partner responsiveness during the first dyadic coping interaction on the three different measures of quality of support provided during the second dyadic coping interaction (i.e., positive dyadic coping, negative dyadic coping, and perceived responsiveness as rated by the partner). First, there was little evidence for a positive effect of perceived responsiveness (T1) on subsequent positive dyadic coping behavior (T2): Individuals who experienced higher levels of partner responsiveness during the first interaction may or may not have been more likely to engage in positive dyadic coping behavior themselves when they were listening to their partner in the subsequent interaction [BF_10_ = 3.57; *B* = 0.07 on the logit scale, 95% credible interval (−0.11, 0.24); Figure 2A]. For negative coping behavior (T2), however, we found strong evidence in favor of a negative effect of perceived responsiveness (T1): Individuals who experienced higher levels of partner responsiveness during the first interaction were less likely to subsequently engage in negative dyadic coping behavior themselves [BF_10_ = 47.19; *B* = −0.33 on the logit scale, 95% credible interval (−0.67, −0.01); Figure 2B]. Finally, there was strong evidence for a positive effect of perceived responsiveness at T1 on subsequent perceived support at T2: Individuals who experienced higher levels of partner responsiveness during the first interaction were rated as more responsive by their partner in the subsequent interaction [BF_10_ = 507.26; *B* = 0.19 on the response scale, 95% credible interval (0.08, 0.30); Figure 2C]. A summary of the Bayes factor analyses for all three studies is given in Table 1, the coefficients are displayed in Figure 3, and the estimated effects are visualized in Figures 2, 4. Additional descriptive statistics for all three studies are provided in Supplementary Tables 1–4.

**TABLE 1:** Bayes factors in favor of lingering effects on the quality of subsequent support provision per study.

	Outcome					
	
	Positive dyadic coping		Negative dyadic coping		Perceived responsiveness (T2)	
			
Predictor	*N*	BF_10_	*N*	BF_10_	*N*	BF_10_
**Perceived responsiveness (T1)**						
Study 1	243	3.57	243	**47.2**	264	**507**
Study 2	129	**151**	129	0.31	121	**271**
**Negative affect**						
Study 1	243	**70.1**	243	**37.0**	264	5.74
Study 2	129	0.15	129	**15.7**	121	1.21
Study 3	342	0.18	342	3.46	–	–

*Bayes factors give the evidence for the model including the relevant predictor [perceived responsiveness (T1); negative affect] versus the null model for each study. Bayes factors printed in bold pass the threshold for substantial evidence in favor of the presence of an effect. Bayes factors are order-constrained based on the hypothesized direction of the effects. Note that perceived responsiveness (T1 and T2) was not measured in Study 3. *N*refers to the number of participants included in each particular analysis.*

#### Spillover Effects of Negative Affect

With regard to the second hypothesis, we assessed the evidence for an effect of residual negative affect after the first dyadic coping interaction (T1) on the quality of support provided during the second dyadic coping interaction (T2). First, the Bayes factor model comparison indicated strong evidence that individuals who experienced higher levels of negative affect after talking about a personal stressor in the first interaction were less likely to engage in positive dyadic coping behavior themselves when they were listening to their partner in the subsequent interaction [BF_10_ = 70.11; *B* = −0.29 on the logit scale, 95% credible interval (−0.57, −0.03); Figure 4A]. Second, these data indicated strong evidence in favor of a positive effect of negative affect on subsequent negative dyadic coping behavior: Individuals who experienced higher levels of residual negative affect after their own sharing interaction were more likely to subsequently engage in negative dyadic coping behavior themselves [BF_10_ = 37; *B* = 0.43 on the logit scale, 95% credible interval (0.00, 0.86); Figure 4B]. Third, the data indicated moderate evidence for a negative effect of residual negative affect on subsequent perceived responsiveness (T2): Individuals who experienced higher levels of negative affect after their own sharing interaction may have been rated as less responsive by their partner in the subsequent interaction [BF_10_ = 5.74; *B* = −0.17 on the response scale, 95% credible interval (−0.35, 0.01); Figure 4C].

## Study 2

Study 2 served as a replication of Study 1. The procedures and materials were almost identical to those of Study 1, with one exception: Participants did not first engage in a conflict interaction. Furthermore, participants were constituted of adolescent and emerging adult (rather than adult) couples, including one female and one male partner.

### Methods

#### Participants and Procedure

A total of 181 couples registered for participation. The data of Study 2 come from a project on romantic relationships in adolescence and emerging adulthood, for which adolescent couples were recruited by means of local newspapers, schools, recreational facilities, and social media. For the purpose of that study, the following eligibility criteria were relevant: Participants had to be (1) in a romantic relationship for a minimum of 1 year; (2) between 16 and 22 years of age; (3) able to read and speak German; (4) and both partners had to agree to participate in the study. This resulted in a total sample of 130 eligible adolescent couples, of which 125 couples were included for the analyses predicting positive and negative dyadic coping and 121 couples for predicting perceived responsiveness. Couples had an average relationship duration of 2.0 years (SD = 1.0). Adolescent females were on average 18.9 years old (SD = 1.6, range = 16.2–22.8 years), and adolescent males were 19.6 years old (SD = 1.6, range = 16.0–22.8 years). Most adolescents were living with their parents (85.2%), and few lived alone (0.8%) or shared an apartment with peers (9.2%). Only 4.8% of the couples cohabited. The study protocol was approved by the ethics committee of the Department of Psychology of the University of Zurich.

#### Materials

##### Negative affect

Before (T0) and after (T1) the first dyadic coping interaction, sharers rated the extent to which they experienced six emotions (i.e., distressed, annoyed, angry, agitated, stressed, sad) on a 5-point Likert scale ranging from 1 (not at all) to 5 (very much). We averaged these items to reflect pre- and post-sharing negative affect.

##### Perceived responsiveness and dyadic coping

Perceived responsiveness (T1 and T2) and positive and negative dyadic coping were measured in an identical way to Study 1.

#### Data Analytic Approach

Statistical models and prior specification were identical to the data analytic approach as specified in Study 1.

### Results

#### Spillover Effects of Perceived Partner Responsiveness

In line with our first hypothesis, the Bayes factor model comparison indicated strong evidence in favor of a positive effect of perceived responsiveness (T1) on subsequent positive coping behavior (T2): Individuals who experienced higher levels of partner responsiveness during the first interaction were more likely to engage in positive dyadic coping behavior themselves when listening to their partner in the subsequent interaction [BF_10_ = 151.38; *B* = 0.36 on the logit scale, 95% credible interval (0.07, 0.63); Figure 2D]. For negative coping behavior (T2), however, there was weak evidence *against* a negative effect of perceived responsiveness (T1): Individuals who experienced higher levels of partner responsiveness during the first interaction were *not* less likely to subsequently engage in negative dyadic coping behavior themselves [BF_10_ = 0.31; BF_01_ = 3.23; *B* = 0.17 on the logit scale, 95% credible interval (−0.33, 0.62); Figure 2E]. It should be noted that the observed effect was in the opposite direction as hypothesized. Finally, in line with our hypothesis, there was strong evidence for a positive effect of perceived responsiveness at T1 on subsequent perceived responsiveness at T2: Individuals who experienced higher levels of partner responsiveness during the first interaction were rated as more responsive by their partner in the subsequent interaction [BF_10_ = 270.66; *B* = 0.26 on the response scale, 95% credible interval (0.11, 0.42); Figure 2F].

#### Spillover Effects of Negative Affect

Contrary to our second hypothesis, there was moderate evidence *against* a negative effect of residual negative affect (T1) on positive dyadic coping (T2): Individuals who experienced higher levels of negative affect after their own sharing interaction were *not* less likely to engage in positive dyadic coping behavior themselves when listening to their partner in the subsequent interaction [BF_10_ = 0.15; BF_01_ = 6.66; *B* = 0.19 on the logit scale, 95% credible interval (−0.15, 0.51); Figure 4D]. Again, it should be noted that the observed effect went in the opposite direction. However, in line with our hypothesis, the data indicated strong evidence in favor of a positive effect of residual negative affect (T1) on subsequent negative dyadic coping behavior (T2): Individuals who experienced higher levels of negative affect after their own sharing interaction were more likely to subsequently engage in negative dyadic coping behavior themselves [BF_10_ = 15.74; *B* = 0.44 on the logit scale, 95% credible interval (−0.11, 1.00); Figure 4E]. Finally, the data indicate no evidence for an effect of negative affect (T1) on subsequent perceived responsiveness (T2): Individuals who experienced higher levels of negative affect after their own sharing interaction may or may not have been rated as less responsive by their partner in the subsequent interaction [BF_10_ = 1.21; *B* = −0.06 on the response scale, 95% credible interval (−0.27, 0.15); Figure 4F].

## Study 3

Study 3 served as a partial replication of Study 1. The procedures and materials were again highly similar to those of Study 1, except that this study did not include any measures of perceived responsiveness and included a different measure of negative affect. Consequently, Study 3 did not allow us to test our hypotheses including perceived responsiveness as a predictor nor as a dependent variable.

### Methods

#### Participants and Procedure

A total of 368 couples participated in Study 3, which constituted the first wave of a multi-wave project examining the impact of stress on the development of (adult) couple relationships. Couples were recruited through advertisements in newspapers and broadcasting. Inclusion criteria were (1) being in a committed relationship for at least 1 year, (2) speaking and understanding German, (3) both partners being willing to participate, and (4) having no records of mental disorders. Three couples did not have observational data (one couple refused to participate in the interaction task, one couple wanted to delete their video after the task, and one video was missing due to technical problems). For two couples, the order of the three interactions was different from the rest of the participants, and their data were therefore excluded from our analyses. Finally, for eight couples, we did not know who was the sharer or the support provider, thereby forcing us to exclude these couples. This yielded a final sample of 355 couples, of which 341 couples were eligible for our analyses (i.e., met our preregistered criteria regarding a maximal number of missing values). Women were on average 46.6 years old (SD = 18.3, range = 19–80 years), and men were 48.5 years old (SD = 18.2, range = 20–82 years). Their relationship duration was on average 21.1 years (SD = 17.3, range = 1–58 years). Most of the couples were married (64.8%; 83.5% was cohabiting). The procedure of the study was identical to that of Study 1. The study protocol was approved by the ethics committee of the Department of Psychology of the University of Zurich.

#### Materials

##### Negative affect

Before (T0) and after (T1) the first dyadic coping interaction, sharers rated their current emotional state on four bipolar dimensions (adapted from Eid et al., 1994): “good mood versus upset,” “placid/serene/relaxed versus irritated/provoked/angry,” “cheerful/happy versus sad/in low spirits,” and “calm/at ease versus stressed/nervous” (scale: 1 = very much, 2 = much, 3 = a little, 4 = a little, 5 = much, 6 = very much). These four items were averaged, with higher scores reflecting greater negative affect.

##### Dyadic coping

Behavioral quality of support during the second dyadic coping interaction (T2) was measured in an identical fashion to Studies 1 and 2, yielding a positive and negative dyadic coping score.

#### Data Analytic Procedure

In Study 3, perceived responsiveness was not measured. Therefore, we only examined the effects of lingering negative affect (T1) on positive and negative dyadic coping (T2). All other details, including the prior specification, were identical to the data analytic approach as specified in Study 1.

### Results

#### Spillover Effects of Negative Affect

Contrary to our second hypothesis, the Bayes factor model comparison indicated moderate evidence *against* a negative effect of residual negative affect (T1) on positive dyadic coping (T2): Individuals who experienced higher levels of negative affect after their own sharing interaction were *not* less likely to engage in positive dyadic coping behavior themselves when listening to their partner in the subsequent interaction [BF_10_ = 0.18; BF_01_ = 5.49; *B* = 0.08 on the logit scale, 95% credible interval (−0.07, 0.23); Figure 4G]. Furthermore, the data indicated little evidence for a positive effect of residual negative affect (T1) on subsequent negative dyadic coping behavior (T2): Individuals who experienced higher levels of negative affect after their own sharing interaction may or may not have been more likely to subsequently engage in negative dyadic coping behavior themselves [BF_10_ = 3.46; *B* = 0.12 on the logit scale, 95% credible interval (−0.17, 0.45); Figure 4H].

## Discussion

### Main Findings

The present set of studies aimed to examine how experiences of one dyadic coping interaction may spill over to affect the dynamics in a subsequent dyadic coping interaction. We hypothesized that the extent to which sharers perceive their partner to have been responsive to their self-disclosure and still carry lingering negative affect shapes their motivation and ability to support their partner when the tables turn and they themselves are put in a situation to provide support. In line with our first hypothesis, sharers who perceived their partner to have been more responsive subsequently engaged in higher-quality support themselves. This enhanced support quality was reflected in partner ratings of perceived responsiveness, as well as observations of positive dyadic coping behavior (though this latter effect was merely weak in Study 1). We did not find consistent evidence for an effect of perceived partner responsiveness on negative dyadic coping behavior. It should be noted that this hypothesis could only be tested in two studies.

The findings regarding our second hypothesis concerning negative affect were mixed. We found no compelling evidence for an effect of higher lingering negative affect on positive dyadic coping behavior or responsiveness as perceived by the partner. Yet, in line with our predictions, higher lingering negative affect did predict an increase in negative dyadic coping behavior in Study 1—an effect that was replicated in Study 2 but only weakly supported in Study 3. Finally, it is worth mentioning that, overall, our effects were not moderated by gender (see Supplementary Table 6), suggesting that the presence (or absence) of spillover effects is similar across men and women.^1^ Taken together, our findings lend support to the notion that perceived partner responsiveness shapes subsequent support quality, though primarily as perceived by the partner. Furthermore, our data suggest that lingering negative affect increases subsequent negative dyadic coping behavior.

### Theoretical and Methodological Implications

It should be noted that a strong floor effect occurred for lingering negative affect, as well as for negative dyadic coping behavior (see Supplementary Figures 1, 2). These floor effects may be partly explained by the videotaped and structured support interactions, which may have led sharers to not fully immerse themselves in the emotional situation as they would be in daily life and support providers to act somewhat socially desirable. Furthermore, throughout our three studies, most couples experienced relatively high relationship satisfaction, which may have partly driven the low occurrence of negative dyadic coping. Even though we took the low frequency of negative dyadic coping into account by running zero-inflated beta regressions, the extremely low variance still reduced the reliability with which our effects could be estimated. Given the prior literature showing that negative affect impairs cognitive functioning (e.g., Curci et al., 2013; Raio et al., 2013) and reduces the motivation to be responsive to one’s partner (e.g., Crocker et al., 2010), it remains possible that negative affect may spill over from one support interaction to the next, impeding the motivation and ability to engage in constructive forms of dyadic coping (see Crocker et al., 2010; Iida et al., 2010). While the current data hint at such effects, they do not allow us to draw firm conclusions.

Our findings suggest that perceiving one’s partner as responsive in turn leads one to be more supportive to one’s partner as well. This is compatible with equity theory, which states that people value fair treatment and therefore are motivated to maintain fairness in their relationships (Walster et al., 1973; Meier et al., 2020). Furthermore, our findings are in line with prior research showing that perceived partner responsiveness predicts an increased willingness to invest in the relationship (Murray et al., 2006), more pro-social behavior toward the partner (Wieselquist et al., 1999), and greater support provision (Lemay and Clark, 2008). These pro-relational behaviors may be explained by the enhanced positive affect, intimacy, and relationship satisfaction that individuals experience as a result of perceived partner responsiveness (e.g., Gable et al., 2006; Debrot et al., 2013; Neal and Lemay, 2014; Lemay and Clark, 2015).

Importantly, the fact that the positive effect of perceived partner responsiveness was most pronounced for subsequent support as perceived by the partner (rather than coded dyadic coping behavior) underlines the important role of perceptions and beliefs regarding others’ responsiveness (see also Uchino, 2009). Prior research shows that these perceptions are partly shaped by the actual responsiveness as enacted by the partner but also substantially biased by motivated interpretation, such as projections of one’s own responsiveness and relationship evaluations (Lemay et al., 2007; Lemay and Clark, 2008, 2015; Maisel et al., 2008; Canevello and Crocker, 2010; Debrot et al., 2012; Neal and Lemay, 2014; Hui et al., 2020). The relatively low correlations between perceived responsiveness and positive and negative dyadic coping behavior observed throughout our studies (see Supplementary Tables 2–4) speak to the subjective nature of these perceptions. Nonetheless, it should be noted that the observed effects of perceived partner responsiveness on subsequent perceived support quality remained qualitatively equivalent when controlling for relationship satisfaction, demonstrating that the observed spillover effects cannot simply be explained by individual differences in relationship quality.

Together, these findings speak to the complex and dynamic nature of dyadic coping interactions, which is mirrored in Reis’s definition of perceived partner responsiveness (Reis et al., 2004). As described by Reis et al. (2004), perceived partner responsiveness is a *process* that is dyadic and thereby cyclical in nature. One partner’s self-disclosure shapes the other’s (ideally responsive) support, which builds trust, elicits reciprocal self-disclosure, and creates intimacy through a bidirectional loop among *both* partners (Wieselquist et al., 1999; Bodenmann, 2005; Cutrona et al., 2007; Rimé, 2009; Finkenauer and Righetti, 2011; Reis et al., 2011; Rossignac-Milon and Higgins, 2018). Furthermore, both the definition and our current findings underline that the emotional and relational consequences of this dyadic process hinge on whether the perceiver *believes* that the response has been understanding, validating, and caring (Reis, 2014; Donato et al., 2015). To the extent that these beliefs are positive, a wealth of personal and relational benefits is brought about (see Lemay and Clark, 2015, for an overview).

Finally, the current findings also have methodological implications. The currently adopted paradigm, which includes several sequential dyadic interactions, is the state-of-the art paradigm used to study both conflict and support interactions in romantic couples. As our findings show, these interactions are not always independent, even though they are usually studied as such. We find that individuals’ lingering emotions and perceptions of their partner’s behavior shape their own behavior in a subsequent interaction both as perceived by the partner and as observed by coders. It may thus be important for future studies to assess participants’ self-reported experiences before and after (and perhaps even during) each interaction (see Sels et al., 2019). Knowing both partners’ (emotional and cognitive) state upon entering a new interaction may contribute to a better understanding of their subsequent behavior.

### Limitations, Strengths, and Future Directions

Several limitations of the present research are worth noting. First, our measures of negative affect were not identical across the three studies, which may partially explain its somewhat inconsistent effect on support quality across studies. Future research may examine the role of specific emotions in spilling over and affecting responsiveness to one’s partner. Different emotions are associated with different appraisals, physiological responses, and behavioral tendencies (Roseman et al., 1994), and these elements may shape the motivation and ability to support one’s partner. For example, anger is typically associated with a social distancing function (e.g., wanting to confront, attack, or criticize another), whereas sadness is typically associated with an affiliative function (e.g., seeking help and support from others; Fischer and Manstead, 2016), which could have opposite effects on subsequent responsiveness to one’s partner. Furthermore, high arousal emotions, such as anger and worry, may impair situational cognitive capacity (and thereby support provision) to a greater extent than low arousal emotions such as sadness or dejection (see Raio et al., 2013). And to come back to our example in the opening of this article, it may be similarly important to separate lingering negative affect that is caused by a stressor external to the relationship (e.g., the dismissal of one’s favorite colleague) from negative affect that is caused by the partner (e.g., perceiving one’s partner as unresponsive; see also Randall and Bodenmann, 2009). While the present set of studies targeted negative affect caused by external stressors, it does not entirely allow distinguishing between these two different sources of negative affect, as part of the lingering negative affect may have been due to perceiving one’s partner as unresponsive. It should be pointed out, though, that our exploratory analyses indicated that the effects of negative affect were independent of perceived responsiveness (see Supplementary Material).

Several other differences between the three studies merit attention. First, in Studies 1 and 3, participants engaged in an 8-min conflict interaction before engaging in the two dyadic coping interactions, whereas in Study 2, participants did not. In this conflict interaction, partners were instructed to talk about a topic that created problems within their relationship and stressed both partners. The three most frequent topics included communication problems with the partner, annoying habits of the partner, and finances. While the presence of a conflict interaction may have caused lingering negative affect experienced toward the partner, we controlled for baseline levels of negative affect prior to the first dyadic coping interaction. Furthermore, given that the pattern of findings is not consistently different between the studies with versus without a preceding conflict interaction, we do not believe this is of concern for the interpretation of the current findings. Second, the samples of the three studies varied in average age, relationship length, and living situation, with Study 2 focusing on adolescents and emerging adults not (yet) cohabiting with their partners and Studies 1 and 3 focusing on adult relationships of varying lengths, in which most partners cohabited. We did not have *a priori* theoretical predictions regarding any potential differences across these samples, and we also did not find any consistently different patterns. Consequently, we consider the use of these three different samples as a strength, allowing us to examine the robustness of our findings.

We examined how experiences of one dyadic coping interaction may spill over to a second dyadic coping interaction by letting partners switch roles as sharer and support provider. In real life, however, these interpersonal dynamics are more complex and involve continuous waves of intrapersonal and interpersonal processes that overlap and interact (Butler and Randall, 2013; Frey et al., 2019). Furthermore, these processes may play out over various time spans including temporally fine-grained dynamics within one conversation (see Frey et al., 2019) but also extended periods that constitute the relational context (see Boiger and Mesquita, 2012). Particularly, potential spillover effects of perceived partner responsiveness form a clear example of how these effects may shape both temporary and more chronic motivations to be a supportive partner. For example, one study showed that day-to-day fluctuations in perceived partner responsiveness were associated with a greater motivation to bond with one’s partner on the same day, as well as on the next day (Iida et al., 2010), which may thus translate into enhanced support provision (e.g., Canevello and Crocker, 2010). Furthermore, repetitive positive or negative sharing interactions with one’s partner likely shape more temporally stable beliefs about one’s partner’s responsiveness, and these (potentially biased) beliefs shape both one’s own support-seeking and support provision behavior (for an overview, see Lemay and Clark, 2015).

Regarding spillover effects of negative affect, we would predict these to be a function of the emotional intensity. Consequently, such spillover is likely to diminish over time, as negative affect typically decreases over time (e.g., Verduyn et al., 2009). While our studies showed spillover effects taking place within minutes, another study showed that negative mood decreased emotional support provision the next day (though it is unclear to what extent negative mood also persisted on the next day; Iida et al., 2010). Furthermore, when negative affect takes the shape of chronic distress, more temporally stable negative effects on support provision are predicted to occur (e.g., Bodenmann et al., 2004; Crocker et al., 2010). Relatedly, it should be noted that spillover effects of negative affect in daily life need not be limited to instances in which sharers disclosed a personally upsetting event but may also be the result of unshared (or perhaps suppressed) negative affect that may subsequently impair support provision. This latter effect is likely to occur frequently in real life: Partners may find themselves in a situation where their own support provision is requested, while not having the motivation or capacity to be responsive due to their own experienced negative affect.

It thus remains an empirical question over what time span these spillover effects play out and what these effects would look like in more real-life contexts. One way of addressing these questions involves examining the microdynamics within one interaction, for example, by using both coded video fragments and self-reports with video-mediated recall (VMR; see Welsh and Dickson, 2005; Kuhn et al., 2017). Another highly fruitful avenue for future research that could shed light on the temporal boundary conditions of potential spillover effects includes examining dyadic coping in people’s daily lives using experience sampling methods (ESMs; see Colombo et al., 2020). Both VMR and ESM studies would additionally allow the examination of within-couple processes that may be different from between-couple processes as targeted in the present article (see Hilpert et al., 2018). Furthermore, by using repeated measures throughout the day that are closer in time to the actual experience, ESM studies enhance the chances of observing naturally occurring emotions. Most importantly, they would also allow testing the dynamic, reciprocal nature of the two partners’ emotions and behaviors continuously impacting one another over time (see Butler and Randall, 2013).

Notwithstanding the limitations and outstanding future research questions, we think the present research is characterized by several strengths. First, we examined support quality in three ways: Couples engaged in two actual, videotaped dyadic coping interactions, allowing us to code their positive and negative dyadic coping behaviors, as well as to obtain partners’ perceptions of the support that they received. As such, our data go beyond classic research that is often limited to self-report, which is crucial given how partner perceptions appear to be highly biased (see Lemay and Clark, 2015). Second and relatedly, our studies explicitly address the inherently dyadic nature of the coping process (Bodenmann, 1995; Bodenmann et al., 2016): Our findings show that one partner’s experiences subsequently shape how responsive the other partner (as well as independent coders) perceive them to be. Finally, we preregistered all our analyses and examined the robustness of our findings in three independent studies using the same methodological paradigm. These studies involved three different samples, thereby representing both adolescent as well as adult couples with varying relationship lengths.

### Concluding Remarks

Throughout three behavioral studies, we showed how the experiences of one dyadic coping interaction may spill over and affect support provision in the next interaction. Our findings lend support for the notion that perceived partner responsiveness shapes subsequent support quality, though primarily as perceived by the partner. Furthermore, our data hint at the potentially detrimental effect of lingering negative affect impairing support provision. Thus, how people feel after sharing their emotions with their romantic partner may impact the way they themselves in turn respond to their partner’s concerns. Together, these findings highlight the dynamic and interdependent nature of dyadic coping. Support interactions are always embedded in the context of the relationship, where partners continuously switch roles as sharer and support provider. Given the importance of dyadic coping for individuals’ emotional and relational well-being (Bradbury and Karney, 2004; Rafaeli and Gleason, 2009; Falconier et al., 2015), obtaining a better understanding into the predictors of helpful (and unhelpful) support is crucial. Our findings speak to a key role of *believing* that one’s partner is responsive to one’s needs in fostering reciprocated responsiveness, which is key in promoting healthy relationship functioning.

## Data Availability Statement

The raw data supporting the conclusions of this article will be made available by the authors, without undue reservation.

## Ethics Statement

The studies involving human participants were reviewed and approved by Department of Psychology, University of Zurich. Written informed consent to participate in this study was provided by the participants’ legal guardian/next of kin.

## Author Contributions

LP and AM developed the study concept and formulated the hypotheses and analysis plan for the preregistrations. FM, VR-A (Study 1), CB, AM (Study 2), and MN (Study 3) were involved in data collection. SH analyzed the data. LP wrote the Introduction, Methods, and Discussion. SH wrote the Results and Supplementary Material. AM, MM, GB, and FM provided valuable feedback on all sections of the manuscript. All authors approved the final version of the manuscript for submission.

## Conflict of Interest

The authors declare that the research was conducted in the absence of any commercial or financial relationships that could be construed as a potential conflict of interest. AM, is co-author on this manuscript.

## Footnotes

1 One exception was observed in Study 3: Women were more strongly affected by lingering negative affect in their own negative dyadic coping behavior than men. However, given that we did not find this moderation effect in Studies 1 and 2, and prior research shows an opposite effect (Bodenmann et al., 2015), we conclude that, overall, there is no compelling evidence for gender differences in spillover effects.
