# Supplementary material for: Spillover Effects When Taking Turns in Dyadic Coping: How Lingering Negative Affect and Perceived Partner Responsiveness Shape Subsequent Support Provision
Source: Front Psychol. 2021 Mar 4;12:637534. doi: 10.3389/fpsyg.2021.637534 (PMC7970130; doi:10.3389/fpsyg.2021.637534)
Supplement: Supplementary file 1 [file Data_Sheet_1.pdf]

Supplementary Material

1 Secondary Analyses

As preregistered, we also ran our models including stress expression of the partner during the second interaction as a covariate, to ensure that the observed effects were indeed due to lingering negative affect and perceived partner responsiveness at T1, above and beyond the second sharer's stress expression at T2. Stress expression was coded using the dyadic coding scheme, and included factual stress expressions, verbally implicit and explicit stress expressions and appeals for problem-focused support. We collapsed across all subcategories, thereby controlling for the total of amount of stress expression. As can be seen in Table S5, the results were qualitatively similar to the results of the main analyses, with two exceptions. A first notable difference included the evidence for the effect of negative affect on negative dyadic coping, which became substantial for Study 3 when controlling for the partner's stress expression (BF_{10} = 10.79 vs. BF_{10} = 3.46). Second, the evidence for the effect of perceived partner responsiveness (T1) on positive dyadic coping in Study 1 actually changed from weak evidence in favor of an effect to weak evidence against an effect (BF_{10} = 0.61 vs. BF_{10} = 3.57).

2 Exploratory Analyses

2.1 Gender Interactions

As preregistered, we exploratorily examined whether any of the hypothesized effects were moderated by gender, such that the nature of the first support interaction might differentially impact support provision in the second interaction for men and women. Given that prior research (Bodenmann et al. 2015) showed that women were better able than men to provide responsive support while having to regulate their own emotional distress, we included gender as a moderator in our main analyses to examine whether any of the potential spillover effects were stronger for men than for women.

As displayed in Table S6, the data indicate no evidence for a moderation effect of gender on the relation between the predictors (perceived responsiveness (T1) and negative affect) and any of the dependent variables. Specifically, there is no evidence that men are more strongly affected by perceived partner responsiveness or lingering affect after their own sharing conversation when subsequently providing support to their partner. Notably, in Study 3, we find strong evidence against an interaction between negative affect and gender on negative dyadic coping in the hypothesized direction (BF_{10} = 0.004) and instead have evidence for an interaction in the opposite direction: Women were more strongly affected by lingering negative affect in their own negative dyadic coping behavior than men (BF_{01} = 274.86).

2.2 Experimental Order

As described in Hypothesis 1 and 2, we predicted the two dyadic coping interactions to be interdependent, such that the nature of the first dyadic coping interaction would impact support provision in the second interaction. Therefore, as pre-registered, we exploratorily tested whether the experimental order (i.e., first or second dyadic coping interaction) had an effect on perceived or behavioral support quality.

As can be seen in Table S7, the data indicated no evidence for order effects in support quality. Support did not differ substantially between the first and second dyadic coping interaction for any of the three operationalizations (i.e., positive dyadic coping behavior, negative dyadic coping behavior, and perceived responsiveness (T2)). Only for perceived responsiveness (T2) in Study 2 we found some evidence in favor of an order effect, yet this was only weak evidence ($BF_{10} = 2.90$). For all other measures and studies, we found evidence *against* an order effect.

2.3 Relationship Satisfaction

To verify that our findings would reflect actual spillover effects, rather than stable individual differences across couples, we reran all our main analyses while controlling for relationship satisfaction. In Study 1, relationship satisfaction was measured with the German version of the CSI-4 ($\alpha_{women} = .83$, $\alpha_{men} = .83$; Funk and Rogge 2007). In Study 2, relationship satisfaction was measured with the German version (Sander and Böcker 1993) of the RAS ($\alpha_{women} = .69$, $\alpha_{men} = .73$; Hendrick 1988). In Study 3, relationship satisfaction was measured with the CSI-4 ($\alpha_{women} = .82$, $\alpha_{men} = .89$), similar to Study 1. Relationship quality was averaged across both partners of each couple. As can be seen in Table S8, the results of the main analyses remain qualitatively equivalent when adding relationship satisfaction as a covariate in the analyses, demonstrating that the observed effects are not simply explained by individual differences in relationship quality.

2.4 Perceived Responsiveness by Negative Affect Interactions

Finally, we conducted an exploratory analysis to investigate whether perceived responsiveness (T1) moderated the effect of lingering negative affect (T1) on dyadic coping and perceived responsiveness (T2), as the effect of negative affect on subsequent support provision might depend on the extent to which the first sharer felt supported themselves. The data, however, provided (slight) evidence *against* an interaction between perceived responsiveness (T1) and negative affect on positive dyadic coping ($BF_{10} = 0.26$ in Study 1; $BF_{10} = 0.62$ in Study 2), on negative dyadic coping ($BF_{10} = 0.23$ in Study 1; $BF_{10} = 0.61$ in Study 2) and on perceived responsiveness at T2 ($BF_{10} = 0.27$ in Study 1; $BF_{10} = 0.73$ in Study 2)[1]. Negative affect thus shaped participants' own subsequent support provision

[1] Note that these Bayes factors were not order-restricted as we did not have clear expectations about the direction of the interaction effect. Additionally, the zero-and-one-inflated beta models failed to converge for the two analyses predicting positive and negative dyadic coping. We therefore decided to use a normal Bayesian beta-regression analysis for these

in a similar manner, regardless of how responsive they had experienced their partner in the previous interaction in response to their own self-disclosure. However, we would like to note that these results should be interpreted with caution, as the perceived responsiveness and lingering negative affect after the first interaction likely are interdependent (e.g., those who perceived high responsiveness may experience lower negative affect due to reduced negative emotions experienced in relation to the stressor).

---

two dependent measures, where we changed dyadic coping scores of 0 to .001 and 1 to .999, in order to satisfy the conditions of a beta-regression.

3 References

Bodenmann, G., Meuwly, N., Germann, J., Nussbeck, F. W., Heinrichs, M., and Bradbury, T. N. "Effects of Stress on the Social Support Provided by Men and Women in Intimate Relationships." *Psychological Science* 26 (October): 1584–94. https://doi.org/10.1177/0956797615594616.

Funk, J. L., and . Rogge, R. D. 2007. "Testing the Ruler with Item Response Theory: Increasing Precision of Measurement for Relationship Satisfaction with the Couples Satisfaction Index." *Journal of Family Psychology* 21: 572–83. https://doi.org/10.1037/0893-3200.21.4.572.

Hendrick, S. 1988. "A Generic Measure of Relationship Satisfaction." *Journal of Marriage and the Family* 50 (February): 93. https://doi.org/10.2307/352430.

Sander, J., and Böcker S. 1993. "Die Deutsche Form Der Relationship Assessment Scale (RAS): Eine Kurze Skala Zur Messung Der Zufriedenheit in Einer Partnerschaft [The German Version of the Relationship Assessment Scale (RAS): A Short Scale for Measuring Satisfaction in a Dyadic Relationship]." *Diagnostica* 39: 55–62.

4 Supplementary Tables and Figures

4.1 Supplementary Tables

Table S1

Means and standard deviations (in brackets) for the main outcome measures and predictors per study

	Study 1	Study 2	Study 3
Positive Dyadic Coping (T2)	0.77 (0.24)	0.58 (0.31)	0.59 (0.26)
Negative Dyadic Coping (T2)	0.02 (0.06)	0.05 (0.10)	0.02 (0.06)
Perceived Responsiveness (T2)	3.56 (0.88)	4.33 (0.68)	–
Perceived Responsiveness (T1)	3.47 (0.93)	4.14 (0.73)	–
Negative Affect (T1)	1.71 (0.66)	1.62 (0.64)	2.57 (0.86)

Note. Positive and negative dyadic coping were proportion scores (0-1), perceived responsiveness (T1 and T2) and negative affect were measured on a 1-5 Likert scale in Study 1 and 2, and negative affect was measured on a 1-6 bipolar scale in Study 3.

Table S2

Correlation matrix Study 1

	Negative Dyadic Coping (T2)	Perceived Responsiveness (T2)	Perceived Responsiveness (T1)	Negative Affect (T1)
Positive Dyadic Coping (T2)	-0.37	0.07	0.05	-0.14
Negative Dyadic Coping (T2)		-0.15	-0.06	0.23
Perceived Responsiveness (T2)			0.25	-0.22
Perceived Responsiveness (T1)				-0.20

Table S3

Correlation matrix Study 2

	Negative Dyadic Coping (T2)	Perceived Responsiveness (T2)	Perceived Responsiveness (T1)	Negative Affect (T1)
Positive Dyadic Coping (T2)	-0.33	0.27	0.20	0.11
Negative Dyadic Coping (T2)		-0.09	-0.09	0.00
Perceived Responsiveness (T2)			0.34	-0.16
Perceived Responsiveness (T1)				-0.26

Table S4

Correlation matrix Study 3

	Negative Dyadic Coping (T2)	Negative Affect (T1)
Positive Dyadic Coping (T2)	-0.21	0.05
Negative Dyadic Coping (T2)		0.07

Table S5

Bayes factors in favor of lingering effects on subsequent support quality per study controlling for stress expressions of the partner

	Outcome		
Predictor	BF_{10} Positive Dyadic Coping	BF_{10} Negative Dyadic Coping	BF_{10} Perceived Responsiveness (T2)
**Perceived Responsiveness (T1)**			
Study 1	0.61	**55.9**	**219**
Study 2	**39.8**	0.27	**134**
**Negative Affect**			
Study 1	**261**	**55.3**	5.54
Study 2	0.61	**18.5**	2.41
Study 3	0.63	**10.8**	–

Note. Bayes factors give the evidence for the model including the relevant predictor (perceived responsiveness (T1); negative affect) versus the null model for each study. Bayes factors printed in bold pass the threshold for substantial evidence in favor of the presence of an effect. Bayes factors are order-constrained based on the hypothesized direction of the effects. Note that perceived responsiveness (T1 and T2) was not measured in Study 3.

Table S6

Bayes factors in favor of spillover effects on subsequent support quality being stronger for men than for women (predictor-by-gender interaction) per study

	Outcome		
Predictor	BF_{10} Positive Dyadic Coping	BF_{10} Negative Dyadic Coping	BF_{10} Perceived Responsiveness (T2)
**Perceived Responsiveness (T1)-by-Gender**			
Study 1	0.46	1.63	0.32
Study 2	0.55	5.75	2.02
**Negative Affect-by-Gender**			
Study 1	0.42	1.90	0.30
Study 2	2.16	1.04	1.51
Study 3	0.28	0.004	–

Note. Bayes factors give the evidence for the model including the relevant interaction (perceived responsiveness-by-gender; negative affect-by-gender) versus the null-model for each study. None of the Bayes factors passed the threshold for substantial evidence in favor of the presence of an effect. Bayes factors are order-constrained based on the hypothesized direction of the effects (i.e., lingering effects are stronger for men than for women). Note that perceived responsiveness (T1 and T2) was not measured in Study 3.

Table S7

Bayes factors in favor of experimental order effects for subsequent support quality per study

	Outcome					
	Positive Dyadic Coping		Negative Dyadic Coping		Perceived Responsiveness (T2)	
	BF_{10}	BF_{01}	BF_{10}	BF_{01}	BF_{10}	BF_{01}
Study 1	0.25	4.03	0.18	5.50	0.16	6.20
Study 2	0.15	6.47	0.19	5.14	2.90	0.34
Study 3	0.49	2.05	0.15	6.89	–	–

Note. Bayes factors give the evidence that the effect of order is not zero, versus that it is zero for each dependent variable and each study. None of the Bayes factors passed the threshold for substantial evidence in favor of the presence of an effect. Note that perceived responsiveness was not measured in Study 3.

Table S8

Bayes factors in favor of lingering effects on subsequent support quality per study controlling for relationship satisfaction

	Outcome		
Predictor	BF_{10} Positive Dyadic Coping	BF_{10} Negative Dyadic Coping	BF_{10} Perceived Responsiveness (T2)
**Perceived Responsiveness (T1)**			
Study 1	1.76	**23.5**	**250**
Study 2	**75.6**	0.10	**50.0**
**Negative Affect**			
Study 1	**164**	**23.7**	7.65
Study 2	0.19	**15.6**	1.16
Study 3	0.14	2.79	–

Note. Bayes factors give the evidence for the model including the relevant predictor (perceived responsiveness (T1); negative affect) versus the null model for each study. Bayes factors printed in bold pass the threshold for substantial evidence in favor of the presence of an effect. Bayes factors are order-constrained based on the hypothesized direction of the effects. Note that perceived partner responsiveness (T1 and T2) was not measured in Study 3.

4.2 Supplementary Figures

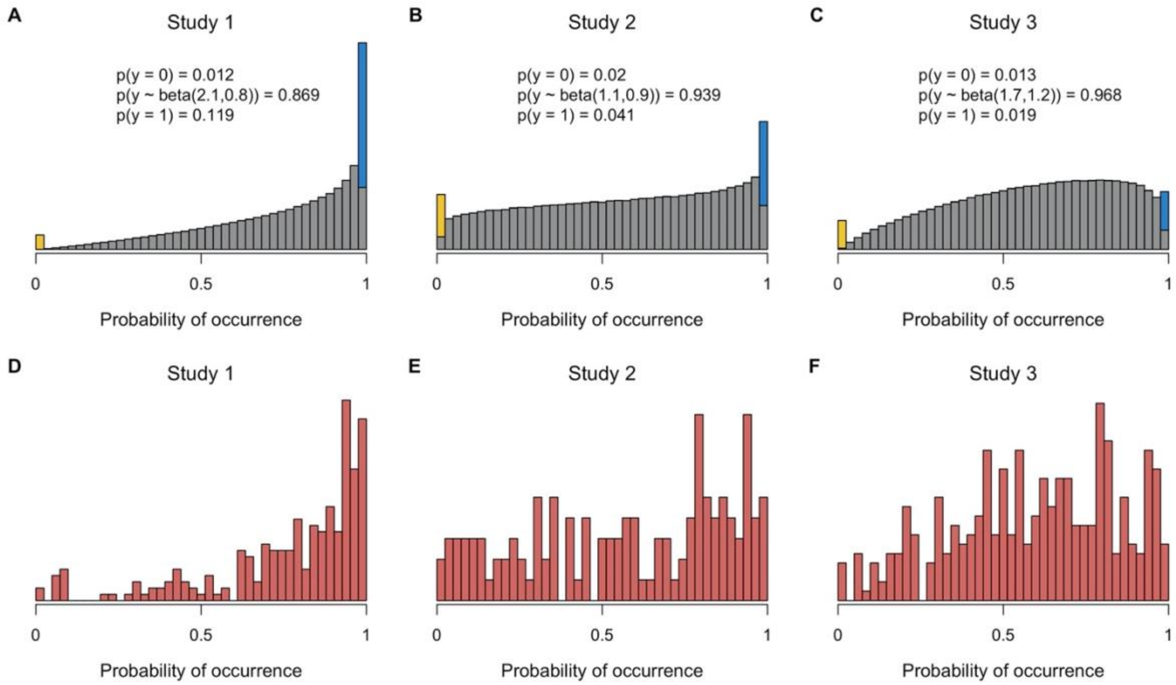

Supplementary Figure 1. Modeled (top panels) and observed (bottom panels) distributions of positive dyadic coping (intercepts) per study. Panels **(A)**, **(B)**, and **(C)** display the modeled zero-and-one-inflated beta distributions that were used to account for the excess ones (behavior exclusively occurred) and zeros (behavior did not occur) in the proportion of observed behavior during the second dyadic coping interaction. Panels **(D)**, **(E)**, and **(F)** show the corresponding frequency distributions of the observed data.

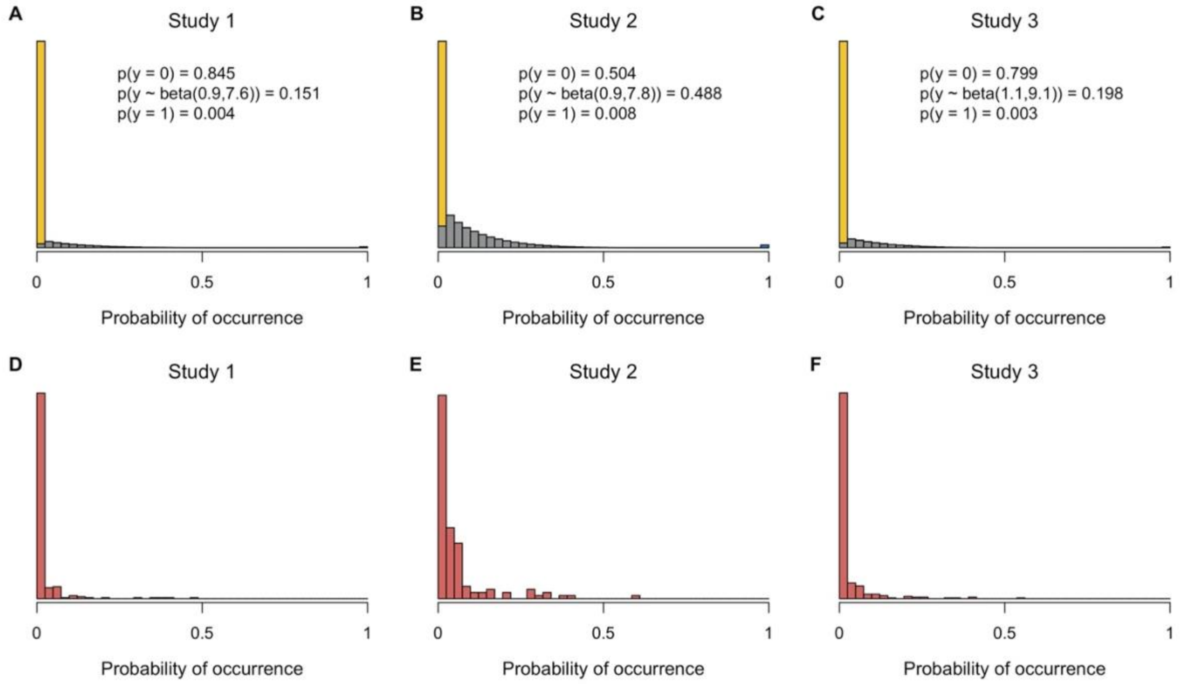

Supplementary Figure 2. Modeled (top panels) and observed (bottom panels) distributions of negative dyadic coping (intercepts) per study. Panels **(A)**, **(B)**, and **(C)** display the modeled zero-and-one-inflated beta distributions that were used to account for the excess zeros (behavior did not occur) in the proportion of observed behavior during the second interaction. Panels **(D)**, **(E)**, and **(F)** show the corresponding frequency distributions of the observed data.
